# The influences of Taiwan's generic grouping price policy on drug prices and expenditures: Evidence from analysing the consumption of the three most-used classes of cardiovascular drugs

**DOI:** 10.1186/1471-2458-8-118

**Published:** 2008-04-12

**Authors:** Chi-Liang Chen, Likwang Chen, Wei-Chih Yang

**Affiliations:** 1 The Department of Accounting, The College of Business, Chung Yuan Christian University, Chung-Li City, Taoyuan County 320, Taiwan; 2 Department of Accounting, College of Management, National Taiwan University, Taipei City 106, Taiwan; 3 Centre for Health Policy Research and Development, National Health Research Institutes, No.35 Keyan Road, Zhunan Town, Miaoli County 350, Taiwan; 4 Institute of Public Health & Department of Social Medicine, School of Medicine, National Yang-Ming University, Taipei City 112, Taiwan

## Abstract

**Background:**

Controlling the growth of pharmaceutical expenditures is a major global challenge. Promotion of generic drug prescriptions or use is gaining increased support. There are substantial contextual differences in international experiences of implementing pharmaceutical policies related to generic drugs. Reporting these experiences from varied perspectives can inform future policy making. This study describes an experience of Taiwan, where patients with chronic (long-term) conditions are usually managed in hospitals and drugs are provided in this setting with costs reimbursed through the National Health Insurance (NHI). It investigates the effects of Taiwan's reimbursement rate adjustment based on chemical generic grouping in 2001. This research also demonstrates the use of micro-level longitudinal data to generate policy-relevant information. The research can be used to improve efficiency of health care resource use.

**Methods:**

We chose the three most-used classes of cardiovascular drugs for this investigation: beta blocking agents, calcium channel blockers mainly with vascular effects, and plain ACE inhibitors. For each drug class, we investigated changes in daily expense, consumption volume, and total expenditures from a pre-action period to a corresponding post-action period. We compared an exposure or "intervention" group of patients targeted by the action with a comparisonor "control" group of patients not targeted by the action. The data sources are a longitudinal database for 200,000 NHI enrolees, corresponding NHI registration data of health care facilities, and an archive recording all historical data on the reimbursement rates of drugs covered by the NHI. We adopted a fixed effects linear regression model to control for unobserved heterogeneity among patient-hospital groups. Additional descriptive statistics were applied to examine whether any inappropriate consumption of drugs in the three classes existed.

**Results:**

The daily drug expense significantly decreased from the pre-action period to the post-action period for the exposure group. The average magnitudes of the decreases for the three classes of drugs mentioned above were 14.8%, 5.8% and 5.8%, respectively. In contrast, there was no reduction for the comparison group. The number of days of the prescription increased significantly from the pre- to the post-action period for both exposure and comparison groups. The total expense also significantly increased for both patient groups. For the exposure group, the average magnitudes of the growth in the total expenditure for the three classes of drugs were 47.7%, 60.0% and 55.3%, respectively. For the comparison group, they were 91.6%, 91.6% and 63.2%, respectively. After the action, approximately 50% of patients obtained more than 180 days of prescription drugs for a six-month period.

**Conclusion:**

The 2001 price adjustment action, based on generic grouping, significantly reduced the daily expense of each of the three classes of cardiovascular drugs. However, in response to this policy change, hospitals in Taiwan tended to greatly expand the volume of drugs prescribed for their regular patients. Consequently, the total expenditures for the three classes of drugs grew substantially after the action. These knock-on effects weakened the capability of the price adjustment action to control total pharmaceutical expenditures. This means that no saved resources were available for other health care uses. Such expansion of pharmaceutical consumption might also lead to inefficient use of the three drug classes: a large proportion of patients obtained more than one day of drugs per day in the post-action period, suggesting manipulation to increase reimbursement and offset price controls. We recommend that Taiwan's government use the NHI data to establish a monitoring system to detect inappropriate prescription patterns before implementing future policy changes. Such a monitoring system could then be used to deter hospitals from abusing their prescription volumes, making it possible to more effectively save health care resources by reducing drug reimbursement rates.

## Background

Controlling the growth of pharmaceutical expenditures is a major challenge all over the world [1-9]. Among various methods for controlling pharmaceutical expenditures, promotion of generic drug prescriptions or use has received much support in recent years [6]. Such promotion is usually through a mechanism of reference pricing or mandatory generic substitution [6]. There are substantial contextual differences in international experiences of implementing pharmaceutical policies related to generic drugs. Reporting experiences under different contexts can inform future policy making.

To this date, there has been limited original research in this area – presumably due to the difficulty in obtaining good data. Most prior studies were conducted in advanced Western countries. Almost all of them used macro-level or aggregate data and most of them suffered difficulties of disentangling the effects of policies concurrently applied to control drug expenditures.

This study describes an experience of Taiwan, where patients with chronic conditions are usually managed in hospitals and drugs are provided in this setting with costs reimbursed through the National Health Insurance (NHI). It investigates the effects of Taiwan's reimbursement rate adjustment based on chemical generic grouping in 2001. This research also demonstrates the use of micro-level data to generate policy-relevant information. This can be used to improve efficiency of health care resource use.

Taiwan started implementing its NHI in 1995. The Bureau of National Health Insurance (BNHI) is the single buyer in this universal public health insurance system. The BNHI established a Pharmaceutical Benefit Scheme (PBS) at the very beginning of the NHI, and since then it has kept adjusting the content of the PBS based on the NHI principles for drug reimbursement listing and pricing [10,11].

The co-payment for drugs is low – with an upper limit of 200 Taiwanese dollars (about 6 US dollars) for each outpatient visit and it is waived in most cases for outpatient care. For instance, there is no co-payment for drugs curing chronic conditions and patients with severe diseases defined by the NHI are exempt from any co-payment for drugs. On the other hand, Taiwan does not have a reference pricing system. The BNHI only reimburses for prescription drugs listed in the PBS [10]. Patients using resources in the NHI system cannot request drugs outside the list by paying price differences.

The high proportion of NHI expenses on pharmaceuticals has always been regarded as a problem in the system. According to the BNHI's estimates, the proportion of NHI expenses on pharmaceuticals was around 25% each year from 1996 to 2002 and about 28% in 2003 [12]. The BNHI uses the methods of positive listing, negative listing, co-payment, and direct regulation for reimbursement rates in order to keep pharmaceutical expenses under control [12]. Direct regulation for reimbursement rates is a primary tool for the bureau to control pharmaceutical spending. Such a strategy mainly targets hospitals as hospital pharmaceuticals represent the primary part of total pharmaceutical expenditures. For instance, in 2004, around 80% of drug expenses for outpatient care were through the hospital sector (estimates based on the NHI research database).

All hospitals in Taiwan can offer outpatient care and have on-site pharmacies. Patients visiting hospitals for outpatient care directly acquire prescription drugs from those hospitals [1]. Most clinics also have on-site pharmacies, and physicians in such clinics prescribe as well as dispense drugs [1]. For each outpatient visit in a clinic, if the prescription duration does not exceed three days, the clinic can use a simplified paperwork to claim a fixed daily charge for the prescription. For outpatient visits with prescriptions of a longer period, claiming drug reimbursements requires paperwork for reporting the details of drugs dispensed. Under this circumstance, physicians in clinics are not interested in dispensing drugs for patients whose prescriptions require the more complicated claim procedure. Consequently, patients with chronic conditions tend to obtain their prescription drugs in hospitals.

Any health care facility can make profit by dispensing prescription drugs as long as its buying prices are lower than the corresponding reimbursement rates [11]. When dispensing drugs, health care facilities collect only co-payments from patients, and obtain the rest of drug charges from the BNHI based on its reimbursement scheme and the fee-for-services pattern. According to the law, the bureau has to regularly adjust reimbursement rates to achieve two goals [11]. The first goal is to reduce price differences among drugs with identical ingredients, content, volume, and dosage form without arousing arguments over intellectual right or health care quality. The second is to make reimbursement rates close to their corresponding weighted average prices in the transaction markets between pharmaceutical companies and health care facilities contracting with the Bureau.

Regarding the first goal, since 2001, the bureau has used the generic grouping method. Drugs with identical ingredient, content, volume, and dosage form are categorized as a group. Within each group, three types of drugs are defined: (1) branded drugs from their original R&D companies, (2) drugs with evidence on effectiveness from bioavailability or bioequivalence studies (BA/BE generics), and (3) generics other than BA/BE generics (common generics) [13].

The BNHI prices branded drugs in reference to prices in ten industrialized countries: UK, France, Germany, Switzerland, Belgium, Sweden, USA, Canada, Australia, and Japan. Branded drugs that have no local BA/BE generics to compete with and new drugs under surveillance cannot be priced over their corresponding median prices in the ten countries. The other branded drugs cannot be priced over 85% of their corresponding median prices in the ten countries.

The prices for BA/BE generics should not exceed 80% of prices for their corresponding branded drugs. A BA/BE generic that is being registered in the PBS for the first time should not be priced higher than the lowest among prices of existing BA/BE generics in the same group. The price of a common generic should not exceed 80%of its corresponding branded drug's price, and should not exceed the lowest price of its corresponding BA/BE generics. For a common generic that is being registered for the first time, its price should be no more than the lowest price of existing common generics in the same group. Compared with most other industrialized countries, Taiwan is generous in pricing generic drugs. This pricing approach is used by the BNHI to motivate health care facilities to dispense generic drugs instead of branded drugs.

While the BNHI allows health care facilities to profit from dispensing drugs, it tries to make drug reimbursement rates close to their corresponding weighted average prices in the transaction market between pharmaceutical companies and health care facilities. For each reimbursement rate adjustment action, the Bureau has to rely on information from a drug market price survey administered to pharmaceutical companies and health care facilities before the action [11]. Pharmaceutical companies and health care facilities cheating in such a survey have legal responsibilities and face punishment from the Bureau, if their frauds are uncovered.

For each drug, the Bureau calculates a weighted mean of market prices using the corresponding transaction volumes as the weights, and then adjusts its reimbursement rate accordingly. Since 2001, there have been three large-scale actions of reimbursement rate adjustments based on chemical generic grouping and market price survey data. They were implemented on April 1 of 2001, March 1 of 2003, and November 1 of 2006, respectively. The 2003 and 2006 actions were introduced under a global budgeting scheme for hospitals, while the 2001 action was not.

Presumably, reimbursement rate adjustment can produce savings for the NHI, and subsequently the BNHI can use the savings for other health services. However, NHI pharmaceutical expenses continue to grow. A major driving force for the growth is increased consumption of pharmaceuticals for treating chronic conditions. It has been argued that Taiwan's expanding population with chronic conditions is a major reason for the quickly increasing consumption. Nevertheless, no research has empirically explored whether any inefficiency in health care resource use is associated with the quickly increasing consumption. To shed light on this issue, this study investigates potentially inefficient use of health care resources following the reimbursement rate adjustment action in 2001.

We used micro-level NHI data to examine drug use before and after the 2001 action. We selected this action in order to focus on effects that directly result from reimbursement rate adjustment, rather than effects mixing influences from both reimbursement rate adjustment and global budgeting. We chose the three most-used classes of cardiovascular drugs based on the first four digits of the coding system of Anatomical Therapeutic Classification (ATC). They are beta blocking agents (ATC code = C07A; called BBAs hereafter), calcium channel blockers mainly with vascular effects (ATC code = C08C; called CCBs), and plain ACE inhibitors (ATC code = C09A; called ACE-inhibitors). In Taiwan, consumption on the three classes accounts for over 50% of the total consumption of cardiovascular drugs (our estimates based on NHI data). The three classes were also among major classes targeted by the 2001 action.

For each drug class, we examined changes in the daily expense, number of days of the prescription, and total expenditures for a pre-action period to a corresponding post-action period. We used longitudinal data for a group of patients who started using drugs of the class in the pre-action period and continued to use such drugs in the post-action period. Following changes in drug consumption for such patients allowed us to investigate hospitals' behaviour with respect to how they influence the irregularly attending patients' drug use patterns after the action, and generate policy-relevant information that can be used to improve efficiency of health care resource use.

## Methods

### Study objectives

For each ATC class of drugs, this study aims to investigate changes in the daily expense, the consumption volume, and total expenditures from a six-month period before the 2001 action (the pre-action period) to a corresponding six-month period after the action (the post-action period) in the hospital setting. This study also intends to examine whether any inappropriately high consumption of drugs in the three classes existed.

### Study periods and populations

The 2001 action was launched on April 1. We selected "September 1,2000 to February 28, 2001" as the pre-action period, and "September 1, 2001 to February 28, 2002" as the post-action period. We did not include the first few months immediately following the action because hospitals in such a period may not show their "planned reactions with respect to the policy change" due to their drug stocks or some of their valid contracts made with pharmaceutical companies previously. The only policy difference between the two study periods was the implementation of the 2001 action. Therefore, this study does not have problems of disentangling effects from policies concurrently applied to control drug expenditures.

Each of Taiwan's generic grouping price adjustment actions was implemented nation wide at the same time. Therefore, we could not conduct a "before-and-after" comparison with a control group by comparing "before-and-after" changes across different places. Alternatively, we compared an exposure group of patients who took drugs targeted by the action, and a comparison group of patients who took drugs not targeted by the action. If the price of the drug a patient obtained from each outpatient visit had a reduced price after the action, we included the patient in the exposure group. If the price of the drug a patient obtained from each outpatient visit was the same or higher after the action, we included the patient in the comparison group. The exposure group was substantially more heavily influenced by the price adjustment than the comparison group in terms of their daily expenses on drugs.

### Hypotheses

In Taiwan, the reimbursement rates for pharmaceuticals covered by the NHI have always been set by the BNHI. There is no variation in reimbursement rates across geographic locations or health care facilities, and all variation across time is due to the BNHI reimbursement policy. For patients in the exposure group, if their physicians did not switch their prescription drugs to other, more expensive drugs after the action, we should observe a reduction in their daily drug expense. However, if their physicians switched their drugs to other more expensive drugs, we might observe no reduction in their daily drug expense. We anticipated that the extent to which physicians could switch their patients' drugs to other more expensive drugs was limited, and thus hypothesized that we would observe a reduction in the daily drug expense for the exposure group. For the comparison group, the physicians had no obvious reasons to switch the prescription drugs to less expensive drugs. We thus hypothesized that we would observe a non-downward trend in the daily drug expense for this patient group.

Regarding consumption volume, we hypothesized that physicians would increase the days of drug prescription for both patient groups, since the NHI drug reimbursement scheme has the fee-for-services pattern. We also hypothesized that the total drug expenditure would increase for both patient groups.

### Unit of analysis

We constructed a longitudinal database for this study. Each patient-hospital group in the database was treated as a cluster and contained two records – one for the pre-action period and the other for the post-action period. This allowed us to apply panel data estimation methods to estimate the influences of the action.

### Data sources

Our first data source is a longitudinal database for 200,000 individuals in Taiwan's National Health Insurance Research Database. The 200,000 individuals are a representative sample of Taiwan's NHI enrollees born in year 2000 or earlier. We linked these data to corresponding registration data of health care facilities in Taiwan and an archive constructed by the BNHI to record all historical data on the reimbursement rates of drugs covered by the NHI.

For each ATC class of drugs, we then constructed a data file consisting of records of outpatient visits made to hospitals for treating hypertension for each aforementioned patient group. Each record contained information about basic characteristics of the hospital and of the patient, the date of the visit, the major ICD 9 (International Classification of Diseases, Ninth Revision) code for this visit, and the prescription duration. Each record also contained information on the name of prescription drug, its corresponding type of price change (down, same, or up) caused by the action, and the drug expense. Based on these visit records, we further constructed a data file comprising patient-hospital-period records. In other words, each record pertained to a patient's use of pharmaceuticals obtained from a specific hospital during the pre-action or the post-action period. The process of constructing data files for this study is described step-by-step in the appendix.

### Dependent and independent variables

The definitions of the three outcome variables for this study are as follows: (1) Dayexp – drug expense per day corresponding to a specific patient-hospital-period record; (2) Totalday – the number of days of the drug prescription corresponding to a specific patient-hospital-period record; (3) Totalexp – the total drug expenditure corresponding to a specific patient-hospital-period record. The dependent variables are"the three outcome variables in the form of the Napierian logarithm."Foreach dependent variable, the explanatory variable is "Post_Action." It was used to capture "the change in the out come variable after the action."

We adopted the logarithm transformation for the outcome variables in order to translate the relationship between an outcome variable and its corresponding explanatory variable into a measure of proportional change [14]. This could facilitate comparisons across different drug classes and across different outcome variables.

### Statistical model and empirical specification

We adopted the fixed effects linear regression model to control for unobserved heterogeneity among patient-hospital groups. The Huber/White/sandwich estimator was employed to obtain robust variance estimates. This method is a commonly used estimator of standard errors, and it is robust without assuming that the standard errors are independent from the explanatory variables and are identically distributed [15]. We used the Stata Statistical Software for our multivariate analysis.

Our empirical specification is as follows:

Y_ij _= β_0 _+ β_1 _Post_Action_ij _+ α_i _+ ε _ij_

The subscript "i" denotes the patient-hospital group, and "j" denotes the period.

For each ATC class of drugs, we estimated separate equations for the exposure group and for the comparison group. We then transformed the coefficient estimate for "Post_Action" into a measure of proportional change, and further calculated the 95% of confidence interval for the proportional change. We compared the confidence intervals between the exposure group and the comparison group to make inferences regarding the influences of the reimbursement rate adjustment action. We also pooled data for both patient groups and estimated an equation for the pooled sample. In this equation, we added an interaction term with respect to the patient group and the period (pre or post) to examine the difference between the two patient groups.

### Descriptive analysis regarding the number of days of prescription, the number of visits, and the number of drug items used

We applied descriptive statistics to depict the distribution of the number of days of drug prescription, the number of visits, and the number of drug items used for both patient groups and for both pre- and post-action periods, respectively. We calculated six selected percentiles for these three measures: 25%, 50%, 75%, 95%, 99% and 100%. These percentiles allowed us to investigate whether any inappropriate consumption of drugs in the three ATC classes existed.

### Sample characteristics

Table 1 summarizes the gender and age distributions of our sample patients. No noticeable differences existed between the two patient groups. We further investigated the characteristics of outpatient visits separately for the two patient groups. Generally speaking, no significant differences existed between the two patient groups in terms of hospital features (ownership, the profit-making type, and the accreditation level), and the hypertension type. Nevertheless, for ACE-inhibitors, a significantly higher percentage of visits were made to public hospitals for the comparison group than for the exposure group. (Due to the length, the characteristics of the outpatient visits were not shown, but are available upon request.)

**Table 1:** Characteristics of hypertension patients

	Exposure group		Comparison group	
		
	N	%	N	%
**Beta blocking agents**				

Number of patients	553	1064	511	

Gender				
Male	280	50.63	258	50.49
Female	273	49.37	253	49.51
Birth year				
1960 or after	16	2.89	21	4.11
1940–1959	219	39.60	224	43.84
1920–1939	294	53.16	248	48.53
1919 or before	24	4.34	18	3.52

**Calcium channel blockers, vascular effects**				

Number of patients	635	1202	567	

Gender				
Male	332	52.28	299	52.73
Female	303	47.72	268	47.27
Birth year				
1960 or after	14	2.20	17	3.00
1940–1959	215	33.86	213	37.57
1920–1939	361	56.85	316	55.73
1919 or before	45	7.09	21	3.70

**ACE inhibitors, plain**				

Number of patients	272	560	288	

Gender				
Male	155	56.99	179	62.15
Female	117	43.01	109	37.85
Birth year				
1960 or after	9	3.31	10	3.47
1940–1959	112	41.18	92	31.94
1920–1939	132	48.53	169	58.68
1919 or before	19	6.99	17	5.90

## Results

### The means of outcome variables

Table 2 summarizes the means of outcome variables for the two patient groups and for the two periods, respectively. The point estimates of the means show that the daily expense significantly decreased for the exposure group after the action, while such a trend was not observed for the comparison group. Both consumption volume (days of drugs prescribed) and total expenditures significantly increased after the action for both patient groups.

**Table 2:** Means of outcome variables

	Pre-action		Post-action		Change after the action
			
	mean	std. dev.	mean	std. dev.	
**Exposure group**					
Beta blocking agents					
Dayexp	11.77	6.79	9.94	6.00	-15.55%
Totalday	98.14	46.32	162.31	55.46	65.39%
Totalexp	1170.62	961.11	1632.12	1141.81	39.42%
Calcium channel blockers, vascular effects					
Dayexp	21.67	7.59	20.21	6.84	-6.74%
Totalday	101.55	45.30	161.79	55.38	59.32%
Totalexp	2211.01	1293.61	3313.68	1641.13	49.87%
ACE inhibitors, plain					
Dayexp	23.16	10.06	21.75	9.25	-6.09%
Totalday	100.21	48.50	154.67	58.88	54.35%
Totalexp	2341.01	1743.67	3374.22	2101.87	44.14%
					
**Comparison group**					
					
Beta blocking agents					
Dayexp	13.07	10.74	13.04	10.10	-0.23%
Totalday	95.03	48.30	160.27	55.68	68.65%
Totalexp	1287.80	1475.68	2117.25	1953.77	64.41%
Calcium channel blockers, vascular effects					
Dayexp	20.49	6.77	20.23	6.70	-1.27%
Totalday	90.44	47.96	159.23	54.86	76.06%
Totalexp	1840.81	1171.14	3259.82	1641.34	77.09%
ACE inhibitors, plain					
Dayexp	23.65	9.05	24.28	10.23	2.66%
Totalday	101.27	48.03	154.38	57.18	52.44%
Totalexp	2383.21	1539.46	3669.49	1994.09	53.97%

### The effects on the daily drug expense

Table 3 indicates that daily drug expenses significantly decreased from the pre-action period to the post-action period for the exposure group. The average magnitudes of decreases for BBAs, CCBs, and ACE-inhibitors were 14.8% (= exp(-0.16)-1), 5.8% (= exp(-0.06)-1) and 5.8% (= exp(-0.06)-1), respectively. In contrast, no reduction in daily drug expenses from the pre- to the post-action period was observed for the comparison group, and there was even an increase of 3.1% (= exp(0.03)-1) for BBAs. These results are consistent with our hypotheses.

**Table 3:** Difference in the daily expense on drugs between the pre- and the post-action periods

	Exposure group		Comparison group	
		
	Coefficient	95% CI of change measured in %	Coefficient	95% CI of change measured in %
Beta blocking agents				
Post_Action	-0.16**	(-17, -12)	0.03^†^	(0, 5)
Test Statistic		F (1,552) = 105.97**		F (1,510) = 3.36^†^
# observations		1106		1022
# patient-hospital groups		553		511
Calcium channel blockers, vascular effects				
Post_Action	-0.06**	(-8, -4)	-0.02	(-4, 0)
Test Statistic		F (1,634) = 26.44**		F (1,566) = 2.49
# observations		1270		1134
# patient-hospital groups		635		567
ACE inhibitors, plain				
Post_Action	-0.06**	(-9, -2)	0.01	(-1, 4)
Test Statistic		F (1,271) = 10.69**		F (1,287) = 0.95
# observations		544		576
# patient-hospital groups		272		288

** p < 0.01; * p < 0.05; ^† ^p < 0.1.

For each ATC class of drugs, the 95% confidence interval of proportional change reveals that change in daily drug expenses from the pre- to the post-action period was significantly different between the two patient groups. Our estimation based on the pooled sample also yielded the same findings (results not shown, but available upon request).

### The effects on the number of days of prescription

The results show a significant increase in the number of days of drugs from the pre- to the post-action period for each patient group (Table 4). Such results are consistent with our hypotheses.

**Table 4:** Difference in the number of days of prescription drugs between the pre- and the post-action periods

	Exposure group		Comparison group	
		
	Coefficient	95% CI of change measured in %	Coefficient	95% CI of change measured in %
Beta blocking agents				
Post_Action	0.57**	(68, 86)	0.61**	(73, 95)
Test Statistic		F (1,552) = 459.97**		F (1,510) = 378.21**
# observations		1106		1022
# patient-hospital groups		553		511
Calcium channel blockers, vascular effects				
Post_Action	0.52**	(60, 76)	0.66**	(83, 106)
Test Statistic		F (1,634) = 418.20**		F (1,566) = 518.10**
# observations		1270		1134
# patient-hospital groups		635		567
ACE inhibitors, plain				
Post_Action	0.50**	(52, 78)	0.47**	(49, 73)
Test Statistic		F (1,271) = 154.52**		F (1,287) = 150.38**
# observations		544		576
# patient-hospital groups		272		288

** p < 0.01; * p < 0.05; ^† ^p < 0.1.

For the exposure group, the average magnitudes of increases for BBAs, CCBs, and ACE-inhibitors were 76.8%, 68.2% and 64.9%, respectively. For the comparison group, the average magnitudes of increases for the three classes were 84.0%, 93.5% and 60.0%, respectively. We found a substantial difference in the magnitude of change between the two patient groups only for CCBs. For this class of drugs, the magnitude of the increase was significantly larger for the comparison group. Our estimation based on the pooled sample reached the same conclusions (results not shown).

### The effects on total expenses

As hypothesized, total expenses significantly increased after the action for both patient groups (Table 5). For the exposure group, the average magnitudes of growth for BBAs, CCBs, and ACE-inhibitors were 47.7%, 60.0% and 55.3%, respectively. For the comparison group, the average magnitudes for these three classes were 91.6%, 91.6% and 63.2%, respectively. The size of increase was significantly larger for the comparison group for both BBAs and CCBs. However, no substantial difference in the size of change emerged between the two patient groups for plain ACE inhibitor. We had the same inferences based on estimation using the pooled sample (results not shown).

**Table 5:** Difference in the total expenditure on drugs between the pre- and the post-action periods

	Exposure group		Comparison group	
		
	Coefficient	95% CI of change measured in %	Coefficient	95% CI of change measured in %
Beta blocking agents				
Post_Action	0.39**	(38, 58)	0.65**	(78, 106)
Test Statistic		F (1,552) = 131.54**		F (1,510) = 316.63**
# observations		1106		1022
# patient-hospital groups		553		511
Calcium channel blockers, vascular effects				
Post_Action	0.47**	(50, 69)	0.65**	(80, 105)
Test Statistic		F (1,634) = 237.32**		F (1,566) = 371.13**
# observations		1270		1134
# patient-hospital groups		635		567
ACE inhibitors, plain				
Post_Action	0.44**	(42, 70)	0.49**	(51, 78)
Test Statistic		F (1,271) = 95.05**		F (1,287) = 139.76**
# observations		544		576
# patient-hospital groups		272		288

** p < 0.01; * p < 0.05; ^† ^p < 0.1.

### The distribution of the number of days of prescription, the number of visits, and the number of drug items used

Table 6 reports the 25^th^, the 50^th^, the 75^th^, 95^th^, 99^th ^and the 100^th ^percentiles for the number of days of drugs, the number of visits, and the number of drug items used. The number of days of drugs prescribed and the number of visits also increased grew considerably after the action. In contrast, changes in the number of drug items were not significant.

**Table 6:** Selected percentiles of the number of days of drugs, of the number of visits, and of the number of drug items

	Exposure group		Comparison group	
		
	Pre-action	Post-action	Pre-action	Post-action
**Beta blocking agents**				

The number of days of drugs				
25%	60	120	56	120
50%	92	180	90	170
75%	140	210	140	210
95%	168	224	168	228
99%	180	252	196	249
100%	210	280	224	300
The number of visits				
25%	2	4	2	4
50%	4	7	4	6
75%	5	7	5	7
95%	6	8	6	8
99%	10	11	8	10
100%	13	14	23	24
The number drug items				
25%	1	1	1	1
50%	1	1	1	1
75%	1	1	1	1
95%	1	2	1	2
99%	2	2	2	3
100%	3	3	2	4

**Calcium channel blockers, vascular effects**				

The number of days of drugs				
25%	60	120	56	120
50%	112	180	90	180
75%	140	210	133	198
95%	168	224	168	225
99%	180	264	182	240
100%	196	330	224	300
The number of visits				
25%	3	5	2	4
50%	4	6	3	6
75%	5	7	5	7
95%	6	8	6	8
99%	12	13	9	11
100%	30	27	12	13
The number drug items				
25%	1	1	1	1
50%	1	1	1	1
75%	1	1	1	1
95%	2	2	1	1
99%	2	2	1	2
100%	4	3	2	3

**ACE inhibitors, plain**				

The number of days of drugs				
25%	60	112	60	112
50%	112	168	104	168
75%	140	202	140	203
95%	168	224	172	224
99%	180	249	196	240
100%	224	288	240	245
The number of visits				
25%	2	4	2	4
50%	4	6	4	6
75%	5	7	5	7
95%	7	9	7	9
99%	12	14	12	13
100%	30	27	12	14
The number drug items				
25%	1	1	1	1
50%	1	1	1	1
75%	1	1	1	1
95%	1	2	1	1
99%	2	3	2	2
100%	3	3	2	2

Approximately 50% of patients' consumption in the post-action period escalated to a level of longer than 180 days of prescription drugs belonging to a specific ATC class for a six-month period. On the other hand, this expansion of pharmaceutical use raised the consumption level of low-volume users in the pre-action period closer to the required level of daily treatment for hypertension after the action.

## Discussion

In Taiwan, patients with chronic conditions do not have to be very cost-conscious for prescription drugs due to the low co-payment policy and because physicians traditionally make decisions on pharmaceutical use for patients. Therefore, the effects of drug reimbursement rate adjustment depend on hospital administrators' and physicians' reactions. This study did not found evidence that hospitals in Taiwan prescribed more expensive drugs to an extent that the policy aim of reducing pharmaceutical unit price was invalid. However, hospitals significantly expanded volumes of prescription drugs for their regularly attending patients after the action. Consequently, total expenditures on the three most-used classes of cardiovascular drugs for such patients dramatically increased.

While the exposure group tended to have a smaller growth rate for total expenditures than the comparison group, the large increases in consumption for the three classes of drugs certainly deserve more attention and scrutiny. Another issue deserving more exploration is that this expansion of pharmaceutical consumption might also lead to inefficient use of the three drug classes, as a large proportion of patients obtained more than one day of prescription drugs belonging to a specific class per day in the post-action period.

Hospitals appeared to ask their patients to return to the hospitals to acquire prescription drugs more frequently. Hospitals in Taiwan usually arrange future visits for their patients, so it is possible that hospitals manipulate time intervals between consecutive visits for their regularly attending patients to expand the volumes of drugs dispensed. Our additional analysis of the number of drug items used for patients obtaining different numbers of days of prescription drugs does not provide evidence that hospitals use "prescribing different drugs in the same class in different out patient visits" as a strategy to be able to dispense more than one day of drugs belonging to a specific class per day and avoid being found fraudulent by the BNHI. To dispense more drugs, hospitals simply increased the number of visits.

Hypertension patients taking medicine more regularly appeared to be a major driver of the growth in expenditures on the three classes of drugs. When we estimated aggregate expenditures on the three drug classes using NHI data for all patients rather than only the sample patients for this study, we found a much lower growth rate of pharmaceutical expenses for each drug class (results not shown). This implies that hospitals targeted patients more regularly taking medicine to expand their revenues from dispensing drugs.

Another issue deserving more attention is that the decrease magnitude of the daily expense found by this study (14.8% for BBAs, 5.8% for CCBs, 5.8% for ACE-inhibitors) was lower than the average level of price reduction for the drugs with reduced prices (16.7% for BBAs, 12.9% for CCBs, 11.4% for ACE-inhibitors – as shown in Table 7). Probably, our sample patients tended to be those taking drugs with smaller price reductions. Another possible explanation for this is that some patients' prescription drugs were switched to more expensive drugs after the action.

**Table 7:** Reimbursement rate changes from the pre- to the post-action period

Beta blocking agents	
Number of all kinds covered by the NHI in the pre- or the post-action period	170
Number of kinds with price reduction after the action	65
% of kinds with price reduction after the action	38.24%
The average level of price reduction (%) of those with reduced prices	16.67%
	
Calcium channel blockers, vascular effects	
Number of all kinds covered by the NHI in the pre- or the post-action period	74
Number of kinds with price reduction after the action	48
% of kinds with price reduction after the action	64.86%
The average level of price reduction (%) of those with reduced prices	12.87%
	
ACE inhibitors, plain	
Number of all kinds covered by the NHI in the pre- or the post-action period	95
Number of kinds with price reduction after the action	50
% of kinds with price reduction after the action	52.63%
The average level of price reduction (%) of those with reduced prices	11.36%

The literature has had only one original research article reporting Taiwan's experience of implementing the generic grouping price policy [12]. This study was based on monthly data of total NHI pharmaceutical expenditures, and used the time-series data intervention analysis method. This paper concludes that the generic grouping price policy shifted the trend of total NHI pharmaceutical expenses, and was helpful for saving health care costs. Nevertheless, data reported in that paper also show that total NHI pharmaceutical expenses kept growing after a significant reduction immediately following the 2001 action. As a result, total expenditures at the end of 2001 were higher than the amount at the end of 2000. Therefore, our findings do not contradict their results.

For other Asian countries with health care systems more similar to Taiwan's, we have not found any original articles reporting their experiences. Some Western countries have experiences related to Taiwan's in respect to reference pricing or mandatory generic substitution. The literature shows mixed results regarding international experiences in reference pricing. Evidence has indicated that reference pricing saved pharmaceutical expenses on angiotension-converting enzyme (ACE) inhibitors and calcium channel blockers in Canada [16], but did not effectively contain public pharmaceutical expenditure for Germany and Spain [17,18]. In Hungary, therapeutic reference pricing increased the average defined daily dose per prescription and expenditure on statins [19]. In contrast, Sweden's experience in mandatory generic substitution policy appears promising, as this policy has produced savings for both the patients and society [20]. In many western countries, in addition to physicians' reactions, the effects of their policies of pricing pharmaceuticals are related to the behavioural changes of pharmacists and patients. In Taiwan, hospital administrators and physicians are main players in reacting to governmental pharmaceutical policies, and pharmacists and patients have negligible roles. Therefore, we cannot directly compare results from this study with those found in these western countries. Nevertheless, experiences in these western countries and Taiwan all make it clear that only relying on a reference pricing system or a generic grouping reimbursement policy cannot effectively control pharmaceutical expenditures.

One primary problem in Taiwan's healthcare sector is that the government has not been able to separate drug prescription and dispensation [1,21]. In Taiwan, physicians have stronger political power than pharmacists and patients. Making profit from dispensing prescription drugs is also traditionally acceptable in the health care sector. Such a tradition originated mainly from a strategy to earn physicians' political support in the period when Japan governed Taiwan and has been kept since [21]. It is a major obstacle to separate drug prescription and dispensation. Moreover, since the NHI payment system is still under much criticism and controversy, the government allows hospitals differentiate between the drug reimbursement rates and hospitals' corresponding purchasing prices as their profits [21]. This certainly gives hospitals incentives to expand the volumes of drugs dispensed and makes it difficult to control pharmaceutical expenditures.

It is certainly politically difficult to fundamentally reform the prescription and dispensation system and the NHI payment scheme in the near future. In the short run, however, the government can construct a monitoring system for studying health care providers' behaviours by taking advantage of the individual longitudinal database that was originally built for handling NHI reimbursements. In Taiwan, the government can always make policies and the non-governmental sectors targeted by these policies can subsequently formulate strategies to make these policies ineffective. To design effective and appropriate policies for controlling health care expenditures, it is necessary to conduct in-depth research regarding health care providers' reactive behaviours for different policy changes.

Micro-level longitudinal data of reliable quality and with sufficient information are necessary for doing good research in this regard. In the least, there should be information regarding health care providers' characteristics and individual patients' demographic background, morbidity conditions and health care utilization. The government could devote more efforts to construct such databases to help researchers conduct more rigorous policy evaluations to generate relevant information for preventing inefficient use of health care resources. In particular, countries with public health insurance should utilize administrative data for reimbursement purposes to construct an information system to enhance their capability of managing health care resource allocation.

This paper has provided a preliminary example of the use of micro-level longitudinal data to uncover inappropriate consumption of drugs. For resource management purposes, it would be even more helpful to trace changes in hospitals' patterns of prescribing and dispensing pharmaceuticals, including their choices within narrow therapeutic classes and volumes of drugs dispensed for different narrow therapeutic classes. Such a tracing system can make it possible to effectively save health care resources and then use saved resources for other health services.

Building a model for such a tracing system based on the NHI research database is a major line of our future research. Investigating the relationships of hospitals' patterns of prescribing and dispensing pharmaceuticals with their characteristics is also a direction of our future research. Further more, we plan to explore whether physicians' behaviour of switching drugs for treating a specific disease affects their patients' use of other health care related to the disease. Since we are not able to acquire data about hospitals' purchasing prices for pharmaceuticals, we cannot directly examine how the amount of profit from dispensing drugs influences hospitals' decision making with respect to drug choices across as well as within narrow therapeutic classes, and volumes of prescription drugs they dispense for different drug classes. This is the major limitation in our research.

## Conclusion

The 2001 price adjustment action, based on generic grouping, significantly reduced the daily expense on each of the three classes of cardiovascular drugs. However, in response to this policy change, hospitals in Taiwan tended to greatly expand the volume of drugs prescribed for their regularly attending patients. Consequently, the total expenditures for the three classes of drugs grew substantially after the action. These knock-on effects weakened the price adjustment action's capability of controlling total pharmaceutical expenditures. This means that no saved resources were available for other health care uses. Such expansion of pharmaceutical consumption might also lead to inefficient use of the three drug classes: a large proportion of patients ended up obtaining more than one day of prescription drugs per day in the post-action period. This suggests manipulation to increase reimbursement and offset price controls. We recommend that Taiwan's government use the NHI data to establish a monitoring system to detect inappropriate prescription patterns before implementing future policy changes. Such a monitoring system could then be used to deter hospitals from abusing their prescription volumes, making it possible to more effectively save health care resources by reductions in drug reimbursement rates.

## Appendix

### The process of constructing the data files for this study

This appendix describes the step-by-step process of constructing the analytical data files. Detailed results generated from each step are not included, but available upon request.

Step 1

We identified outpatient visits based on the following criteria, for each ATC class of drugs:

1. those made by all hypertension patients in the NHI database of 200,000 enrolees.

2. those made in the pre- or the post-action periods (the pre-action period: 2000/09/01 to 2001/02/28; the post-action period: 2001/09/01 to 2002/2/28).

3. those with a major ICD code of 401–405.

4. those with a prescription including the specific ATC type of drug.

Step 2

We retained data of those visits that included prescriptions of only one drug belonging to the ATC class. (Almost all visits belonged to this type.) This was done to simplify the analysis without losing essential data for making inferences.

Step 3

1. For each visit, we combined information regarding the patient identification number and the hospital identification number to create a group identification (ID) number (These numbers were scrambled so that the real identity of the patient was kept confidential).

2. We defined four types of visits with: (1) a patient-hospital group ID number appearing in both the pre- and the post-action periods for individuals with only 1 ID; (2) a patient-hospital group ID number appearing in both the pre- and the post-policy periods for individuals with more than 1 ID; (3) a patient-hospital group ID number appearing only in the pre-action period; (4) a patient-hospital group ID number appearing only in the post-action period. Individuals paying type 3 or type 4 visits used the drug only in either the pre- or the post-action periods, or they switched hospitals after the action. This study focuses on how hospitals reacted to the action by influencing their regular patients' drug usage; therefore, analysis with respect to the last two types of patients was not conducted.

Step 4

1. We removed data of type 3 and 4 visits for the purpose of comparing outcomes between the two periods for each patient-hospital group.

2. We kept only data of type 1 visits for the sake of simplifying the analysis without losing essential data for making inferences (Almost all visits belonged to this type).

Step 5

We removed data of the individuals who did not belong to the exposure or the comparison group, and data of those who had missing variables (The percentages of visits dropped after this step ranged from 8 to 9%. The percentages of patients dropped after this step ranged from 13 to 16%).

Step 6

We constructed the following data files for this study.

(1) Data file 1 (for beta blocking agents, patient-hospital-period records): Number of observations = 2128 (number of total patient-hospital groups × 2: one for the pre-action period, and one for the post-action period).

(2) Data file 2 (for calcium channel blockers mainly with vascular effects, patient-hospital-period records): Number of observations = 2404.

(3) Data file 3 (for plain ACE inhibitors, patient-hospital-period records): Number of observations = 1120.

## Competing interests

The author(s) declare that they have no competing interests.

## Authors' contributions

C-LC designed the study, conducted data analysis, and participated in drafting the manuscript. LC participated in the study design and data analysis and took primary responsibility for drafting the manuscript. W-CY provided substantial help in data analysis and manuscript preparation. All authors have read and approved the manuscript.

## Pre-publication history

The pre-publication history for this paper can be accessed here:


